# Synergistic Enhancement of Photodynamic Cancer Therapy with Mesenchymal Stem Cells and Theranostic Nanoparticles

**DOI:** 10.1021/acsami.4c10098

**Published:** 2024-09-10

**Authors:** Greta Butkiene, Aleja Marija Daugelaite, Vilius Poderys, Riccardo Marin, Simona Steponkiene, Evelina Kazlauske, Ilona Uzieliene, Dainius Daunoravicius, Daniel Jaque, Ricardas Rotomskis, Artiom Skripka, Fiorenzo Vetrone, Vitalijus Karabanovas

**Affiliations:** † Biomedical Physics Laboratory of the National Cancer Institute, P. Baublio St. 3b, Vilnius LT-08406, Lithuania; ‡ Faculty of Medicine, Vilnius University, M. K. Ciurlionio g. 21, Vilnius LT-03101, Lithuania; § Nano for Bioimaging Group (nanoBIG), Departamento de Física de Materiales, Facultad de Ciencias, Universidad Autónoma de Madrid, Madrid 28049, Spain; ∥ Institute for Advanced Research in Chemical Sciences (IAdChem), Universidad Autónoma de Madrid, Madrid 28049, Spain; ⊥ Department of Chemistry and Bioengineering, Vilnius Gediminas Technical University, Sauletekio Ave. 11, Vilnius LT-10223, Lithuania; # Department of Regenerative Medicine, State Research Institute Centre for Innovative Medicine, Santariskiu g. 5, Vilnius LT-08406, Lithuania; ¶ Clinicus Vilnius, V. Grybo g. 17-135, Vilnius LT- 10318, Lithuania; ∇ Nano for Bioimaging Group (nanoBIG), Instituto Ramón y Cajal de Investigación Sanitaria (IRYCIS), Hospital Ramón y Cajal, Madrid 28034, Spain; ○ Biophotonics Group, Laser Research Center, Physics Faculty, Vilnius University, Sauletekio Ave. 9, Vilnius LT-10222, Lithuania; ⧫ Centre Énergie, Matériaux et Télécommunications, Institut National de la Recherche Scientifique (INRS), Université du Québec, Varennes, Québec J3X 1P7, Canada; †† Centre Québécois sur les Matériaux Fonctionnels (CQMF)/Quebec Centre for Advanced Materials (QCAM), Montréal, Québec J3X 1P7, Canada

**Keywords:** mesenchymal stem cells, tumor homing, decoupled theranostics, upconverting nanoparticles, nanomedicine, drug delivery

## Abstract

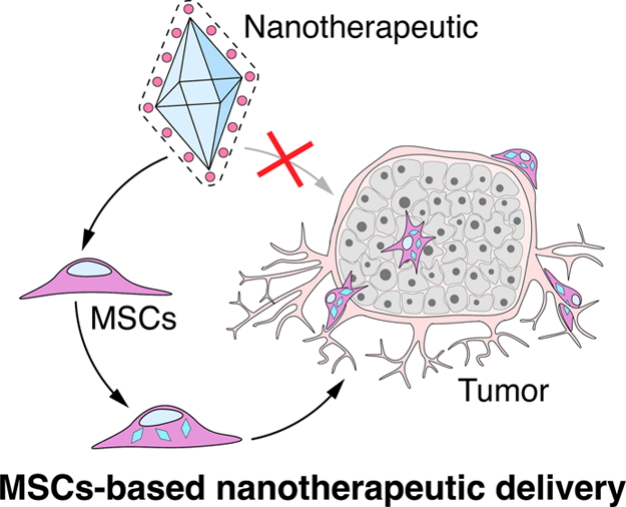

Nanoparticles engineered to combat cancer and other life-threatening diseases may significantly improve patient outcomes. However, inefficient nanoparticle delivery to tumors limits their use and necessitates the development of complex delivery approaches. Here, we examine this issue by harnessing the tumor-homing abilities of human mesenchymal stem cells (MSCs) to deliver a decoupled theranostic complex of rare earth-doped nanoparticles (dNPs) and photosensitizer chlorin e6 (Ce6) to tumors. We show that both bone-marrow- and skin-derived MSCs can transport the dNP-Ce6 complex inside tumor spheroids, which is challenging to accomplish by passive delivery alone. MSCs deliver the dNP-Ce6 complex across the tumor spheroid, facilitating more effective photodynamic damage and tumor destruction than passively accumulated dNP-Ce6. The dNP-Ce6 complex also provides the built-in ability to monitor the MSC migration without causing undesired phototoxicity, which is essential for maximal and side-effect-free delivery of nanoparticles. Our results demonstrate how MSCs can be used as delivery vehicles for the transportation of the dNP-Ce6 complex, addressing the limitations of passive nanoparticle delivery and providing light-based theranostics.

## 1 Introduction

Cancer is a complex and challenging disease. Among other issues, commonly prescribed chemotherapy drugs have low tumor selectivity, often leading to poor survival rates and long patient recovery times.^1^ To overcome the challenges of conventional therapeutics, innovative solutions have arisen from the development of nanoparticles (NPs) designed to bypass biological barriers and selectively destroy tumors.^2^ However, despite significant advances in nanotechnologies, NP delivery and penetration into tumors is still low: less than 1% of intravenously injected NPs reach the cancer^3^ It is now understood that passive delivery of NPs via the enhanced permeability and retention effect,^4−7^ active targeting with antibodies,^8,9^ or even direct injection of NPs into tumors^10−12^ has unsatisfactory specificity, accumulation, and distribution in cancerous tissues. Thus, novel delivery approaches are needed for credible bench-to-bedside translation of NPs.

To overcome the pitfalls of NP delivery, we investigated mesenchymal stem cells (MSCs) as nature-inspired vehicles to deliver cancer therapeutics. In the body, MSCs are located within specific niches and, upon receiving cytokine or growth factor signals, they migrate toward a wound or other inflammation sites, including cancerous tumors.^13,14^ Healthy tissues do not secrete MSC-attracting signals, which makes MSCs selective to the damaged tissues. Using their tumor-homing properties, MSCs can efficiently target and infiltrate tumor sites. Thus, MSCs could be carriers for antitumor drugs, therapeutic genes, or proteins.^15,16^ Such tumor-homing properties of MSCs can be exploited to transport NPs to tumors with enhanced selectivity, delivering more NPs for greater antitumor activity.^17−20^ Despite promising early results, it remains unclear how the tumor-targeting abilities of MSCs depend on their tissue of origin, e.g., bone marrow vs muscle, skin, or placenta. Bone marrow MSCs (BM-MSCs), including human BM-MSCs, are the most used type; however, their isolation procedure is complex and painful for donors, spurring research on new sources of MSCs. As an alternative source, MSCs could be harvested from excess skin that remains after plastic surgery, which is easily accessible and donor-friendly.^20^

Several considerations are crucial for effectively transporting NPs via MSCs to the tumor and initiating the treatment. First, a sufficient number of NPs should accumulate in MSCs without causing adverse cytotoxicity to the carrier cells themselves. Second, MSCs loaded with NPs should retain their tumor-homing and tropic properties, and NPs should retain their properties inside the cells. Ideally, it is desirable to monitor the migration of MSCs toward the tumor using optical or magnetic resonance imaging. Third, only upon appropriate stimulus must the NP cargo be released from MSCs into the tumor microenvironment, ultimately eradicating the tumor. Rare earth (RE^3+^)-doped nanoparticles (RENPs), which combine therapy and diagnostics (theranostic) functionalities but can execute them independently, could fulfill the above conditions and are ideal cargo for MSCs.

Specific combinations of RE^3+^ dopants in RENPs convert long-wavelength near-infrared (NIR) excitation into emission at shorter (i.e., upconversion) or longer (i.e., downshifting) wavelengths. Upconversion emission can be used for on-demand activation of light-based therapies,^21^ such as photodynamic therapy (PDT). In upconversion-assisted PDT, RENPs act as energy donors for classic photosensitizers. This generates reactive oxygen species (ROS) subcutaneously, allowing for the treatment of deep-seated tumors that are inaccessible to short-wavelength direct excitation of photosensitizers.^21^ Meanwhile, NIR-to-NIR downshifting photoluminescence of RENPs in the NIR optical transparency windows (e.g., NIR-II, spanning from 1000 to 1800 nm) is suitable for deep-tissue imaging and diagnostics.^22,23^ Importantly, core/shell engineering of RENPs affords orthogonal excitation of upconversion or downshifting emissions, which results in RENPs with decoupled therapeutic and imaging properties.^24^

**Scheme 1:**
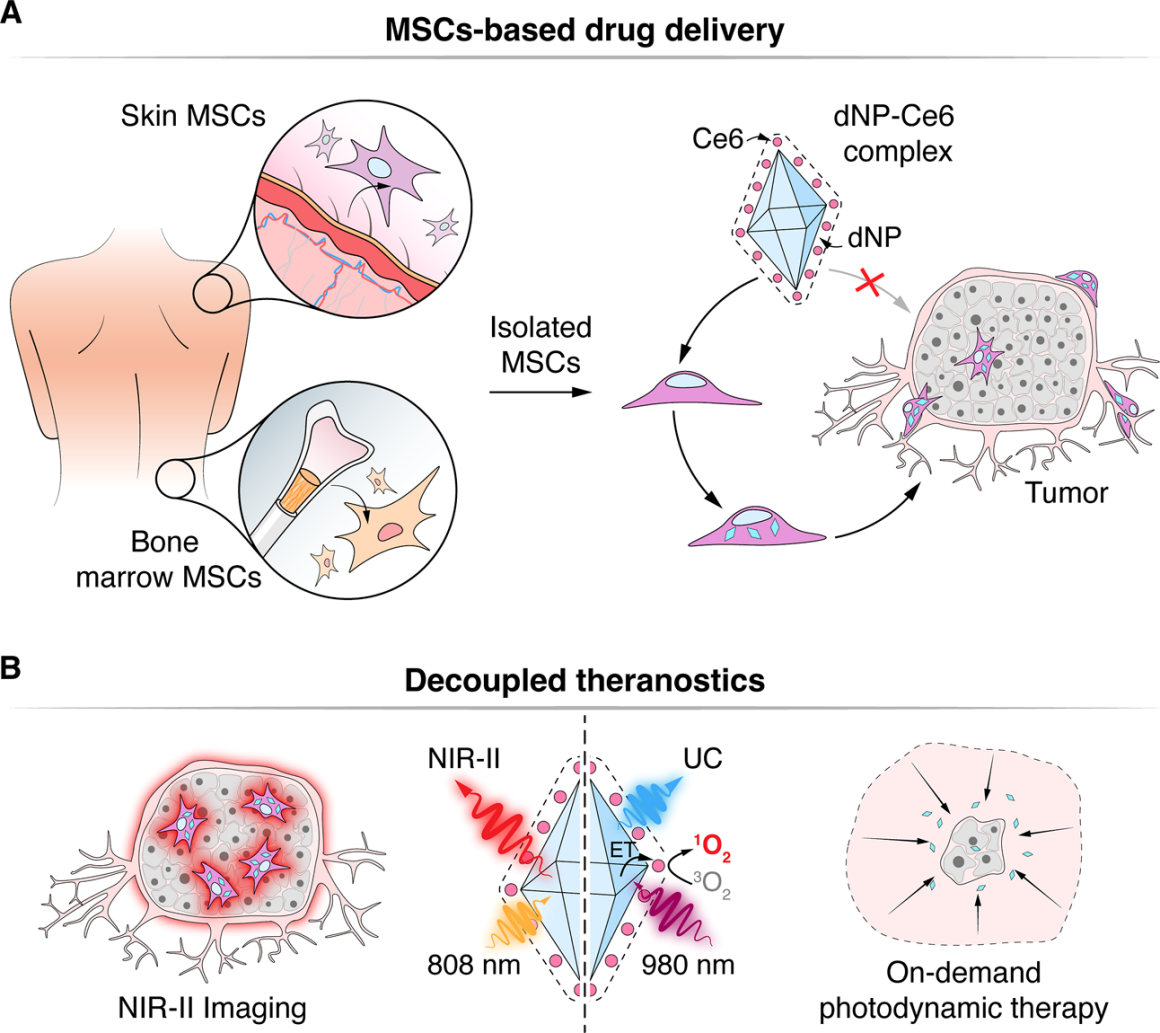
(A) Schematic Illustration of MSCs Isolated from Skin or Bone Marrow, Their Loading with dNP-Ce6 Complex, and Delivery to the Tumor (B) the use of the dNPp-Ce6 complex enables decoupled theranostics. The migration of MSCs could be monitored by NIR-II emission excited at 808 nm without adverse phototoxicity (left). In contrast, tumor eradication could be accomplished by producing cytotoxic oxygen species solely with 980 nm excitation (right). MSCs–mesenchymal stem cells, dNPs–decoupled theranostics nanoparticles, Ce6–chlorin e6, UC – upconversion, ET – energy transfer.

Here, we investigate how skin-derived MSCs (S-MSCs) and BM-MSCs can be used to transport a complex of decoupled theranostic RENPs (dNPs) and the photosensitizer chlorin e6 (dNP-Ce6) (Scheme 1). We found that S-MSCs could penetrate and internalize into cancer cell spheroids as effectively as BM-MSCs and that both cell types prefer to migrate toward the tumor but not the other used chemoattractant. Moreover, the dNP-Ce6 complex enhances the migratory capacity of MSCs, resulting in a higher number of migrated cells toward cancer cells than MSCs without the complex. After delivering the dNP-Ce6 complex to cancer cells, the complex can destroy cancer after two doses of NIR irradiation. The first dose of irradiation damages MSCs and releases the dNP-Ce6 complex into the tumor microenvironment to reaccumulate in cancer cells. The second dose of irradiation destroys cancer cells and shrinks the tumor spheroid. Our study exemplifies how tumor-homing MSCs can improve tumor therapies, facilitating the selective and specific delivery of rationally designed nanotherapeutics.

## 2 Results and Discussion

### 2.1 Properties of the dNP-Ce6 Complex

**Figure 1:**
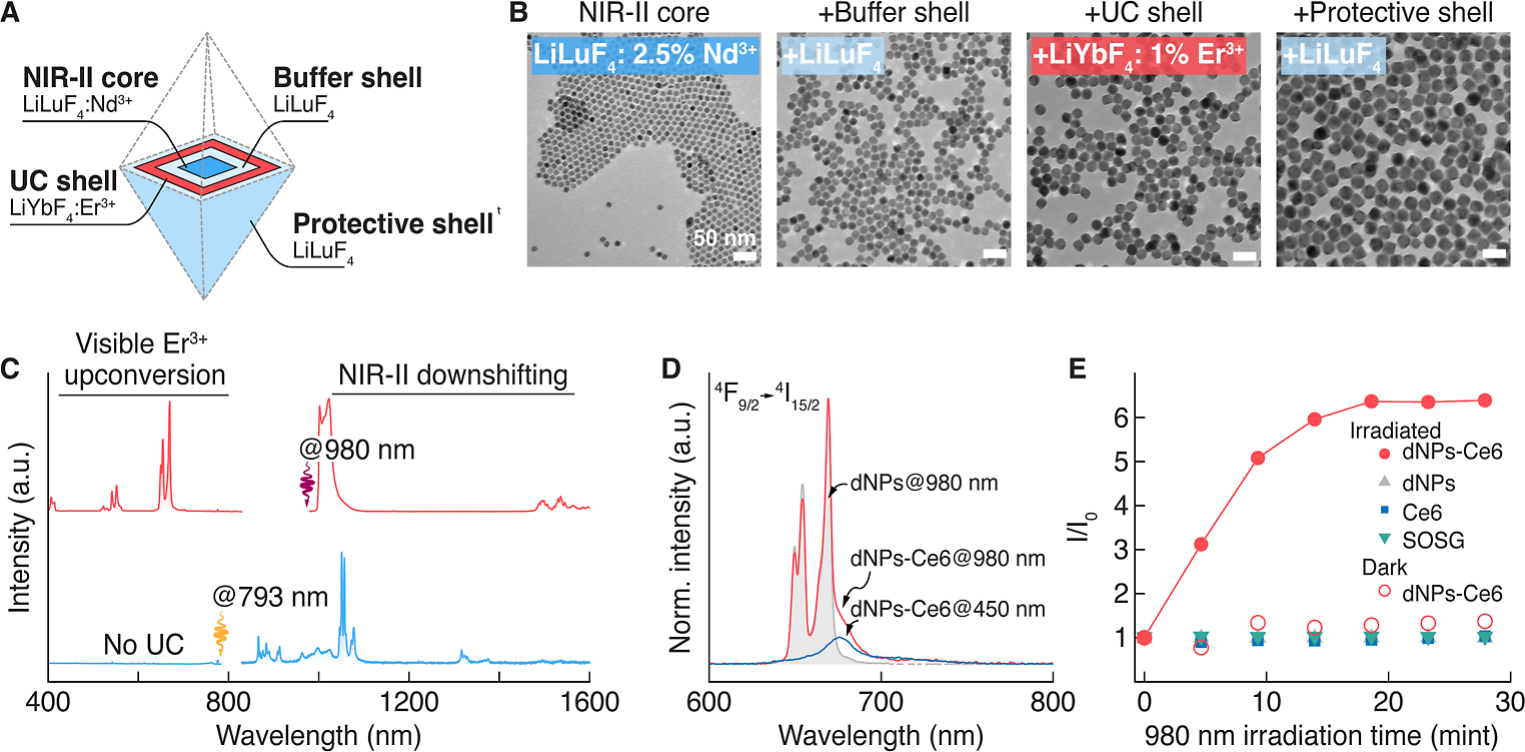
(A) Schematic representation of core/multishell LiLuF_4_: Nd^3+^/LiLuF_4_/LiYbF_4_: Er^3+^/LiLuF_4_ dNPs. (B) TEM micrographs of dNPs at each step of their growth. (C) Visible upconversion and NIR downshifting emission of dNPs under 793 and 980 nm excitation (laser power densities of 100 W/cm^2^). (D) Upconversion emission of dNPs before (gray shaded spectrum) and after being coencapsulated with Ce6 (red spectrum). The spectrum of the dNP-Ce6 complex under 450 nm LED irradiation (blue spectrum) shows Ce6 emission when excited directly. (E) 980 nm irradiation dose-dependent production of ROS (e.g., singlet oxygen) by the dNP-Ce6 complex, dNPs, Ce6, and SOSG.

To prepare a multifunctional nanotherapeutic platform, we synthesized dNPs with a core–shell architecture that enables orthogonal excitation of Er^3+^ upconversion or Nd^3+^ downshifting emission when exciting with different NIR lasers (Figure 1A). Adapting a previously reported procedure,^25^ we synthesized LiLuF_4_: 2.5 mol % Nd^3+^ core NPs (NIR-II core) on which shells were grown: intermediate undoped LiLuF_4_ buffer shell, LiYbF_4_: 1 mol % Er^3+^ upconversion shell, and outer undoped LiLuF_4_ protective shells (Figure 1B; see Experimental Section for details). An intermediate LiLuF_4_ buffer shell was used to eliminate energy transfer between the NIR-II core and upconversion shell,^26−28^ while the outer shell reduced surface quenching.^29^ Transmission electron microscopy (TEM) and powder X-ray diffraction (XRD) analysis showed that the dNPs exhibited a small size (33.5 ± 1.7 × 30.8 ± 1.4 nm) and tetragonal *I*4_1_/*a* phase (Figures S1 and S2).

To validate the orthogonal excitation of dNPs, we characterized their luminescence by using lasers of different wavelengths. Under 980 nm excitation, we observed characteristic Er^3+^ upconversion emission in the visible spectral range with a dominant red emission line at 660 nm (Figure 1C). Notably, we did not detect visible upconversion emission under 793 nm excitation (corresponding to Nd^3+4^I_9/2_ → ^4^F_5/2_ transition in the 790–810 nm range), and the emission spectrum of dNPs consisted entirely of Nd^3+^ lines at 880, 1064, and 1320 nm. These results confirm the orthogonal excitation design of the dNPs, wherein visible upconversion (intended for therapy) is initiated with a 980 nm laser, and NIR downshifting emission (intended for imaging) is observed uniquely under 793/808 nm excitation.

Subsequently, we encapsulated the dNPs in an amphiphilic polymer coating (see Materials for details), which rendered dNPs to be dispersed in water and enabled interaction with Ce6 molecules, forming the dNP-Ce6 complex.^30,31^ We spectrally characterized the obtained dNP-Ce6 complex with a 980 nm laser and observed the Ce6 fluorescence at 670 nm, suggesting that dNPs excite Ce6 via energy transfer (Figure 1D; red spectrum). Direct excitation by a blue LED (450 nm) confirmed the assignment of the fluorescence band at 670 nm to Ce6 molecules (Figure 1D; blue spectrum); when Ce6 is located in a hydrophobic environment, like an inner part of an amphiphilic polymer, its fluorescence shifts from 662 to 670 nm.^32,33^

Subsequently, we used a singlet-oxygen sensor green (SOSG) assay to assess the efficacy of the dNP-Ce6 complex in generating ROS under therapeutic 980 nm irradiation. Indirect Ce6 activation via 980 nm-excited dNPs affords deeper light penetration into tissues,^21,34^ allowing us to reach deep-seated tumors and expand the application of photodynamic therapy. Efficient ROS production from the dNP-Ce6 complex was measured after 30 min of 980 nm laser exposure (Figure 1E, red-filled circles). No ROS generation was detected from the dNP-Ce6 complex in the dark or when solutions of dNPs, Ce6, or SOSG were irradiated. Overall, we could affirm that the dNP-Ce6 complex has the desired optical properties to be used as a theranostic agent in the context of MSC-based tumor therapy.

### 2.2 Cellular Uptake and Biocompatibility of the dNP-Ce6 Complex

**Figure 2:**
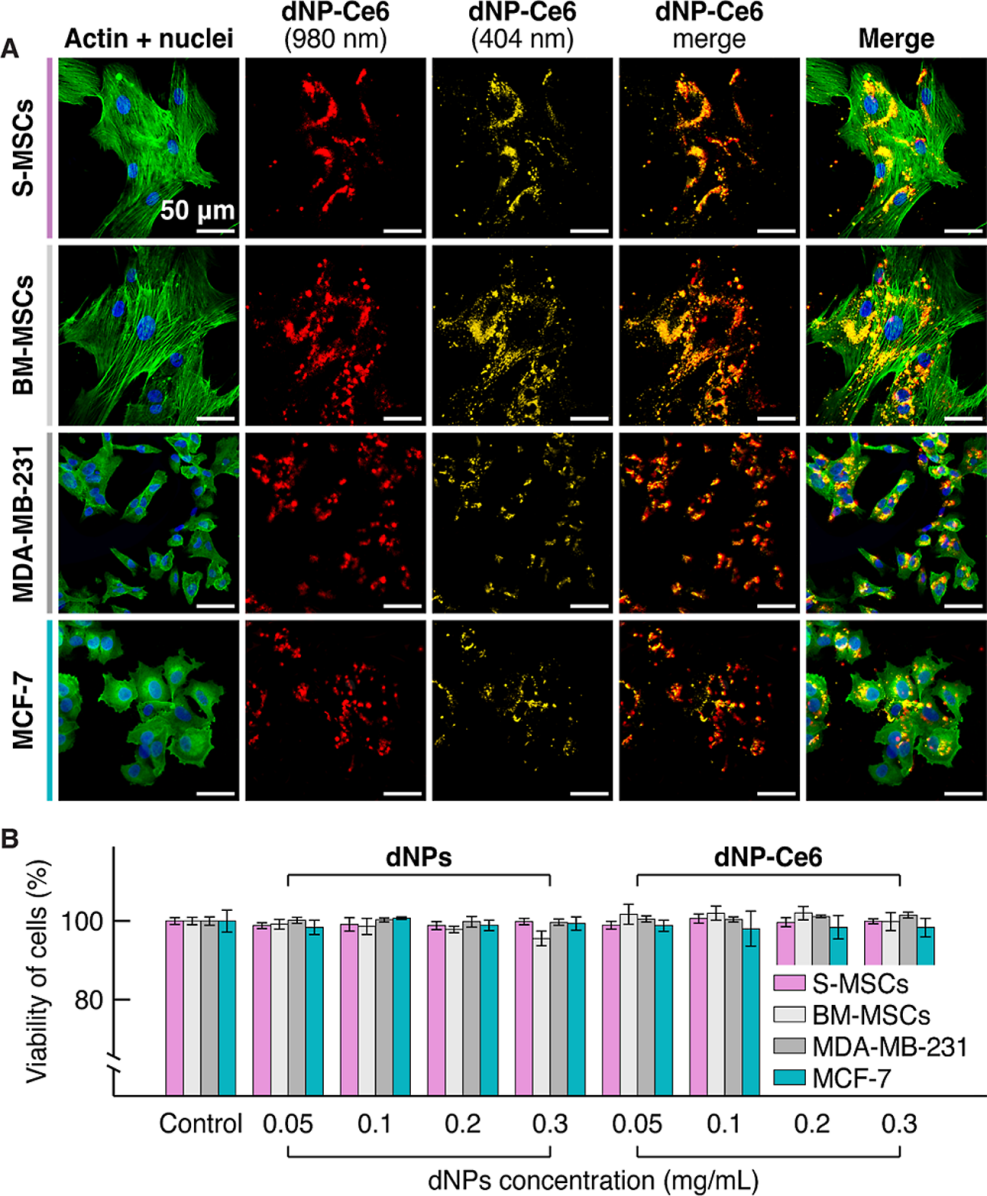
Intracellular accumulation and cytotoxicity of dNPs and the dNP-Ce6 complex. (A) Confocal microscopy images of the S-MSCs, BM-MSCs, MDA-MB-231, and MCF-7 cells, incubated with the dNP-Ce6 complex (0.1 mg/mL, 24 h incubation). Blue – nuclei stained with Hoechst (λ_ex_ = 404 nm); green – F actin filaments stained with Alexa488 Phalloidin dye (λ_ex_ = 488 nm); red – dNP-Ce6 complex (λ_ex_ = 980 nm); and yellow – dNP-Ce6 complex (λ_ex_ = 404 nm). Scale bar −50 μm. (B) The viability of S-MSCs, BM-MSCs, MDA-MB-231, and MCF-7 cells after 24 h incubation with dNPs or the dNP-Ce6 complex in the dark. No statistically significant differences were found at *p* ≤ 0.05. Error bars represent the standard deviation.

Considering the use of the dNP-Ce6 complex in stem-cell-based tumor therapy, we evaluated the cytotoxicity and uptake efficacy of the complex in S-MSCs, BM-MSCs, and cancerous MCF-7 and MDA-MB-231 cells. Cellular accumulation studies were performed after incubation for 24 h with the dNP-Ce6 complex (0.1 mg/mL), and the fixed cells were imaged by laser scanning confocal microscopy. We observed the dNP-Ce6 complex inside different cells, most prominently in the perinuclear region of the cytoplasm (Figure 2A). We note that the dNP-Ce6 complex remained intact after cellular uptake, as confirmed by the colocalized emissions of dNPs (λ_ex_ = 980 nm) and Ce6 (λ_ex_ = 404 nm) in laser scanning confocal microscopy images (Figure 2A).

Having confirmed that the dNP-Ce6 complex can be taken up by different cells, we evaluated the biocompatibility of the dNPs and the dNP–Ce_6_ complex since the NPs cargo should be nontoxic to MSCs when no external stimulus that triggers singlet oxygen generation is applied. S-MSCs, BM-MSCs, MDA-MB-231, and MCF-7 cells were incubated with dNPs or the dNP-Ce_6_ complex (0.05, 0.1, 0.2, and 0.3 mg/mL) for 24 h, and cell viability was measured via a CyQUANT LDH cytotoxicity assay. We observed no statistically significant effect on the cell viability after 24 h of incubation, and all investigated cells maintained above 96% viability (Figure 2B). These results underline that the dNP-Ce6 complex (and dNPs) has no dark toxicity (without laser irradiation) and is biocompatible with MSCs in the range of the tested concentrations.

### 2.3 MSC Migration Studies

Selecting MSCs with the best migration properties for effective stem-cell-based therapy is essential. BM-MSCs are the most studied phenotype of MSCs; however, their application in the clinic can be met with resistance due to complex and painful isolation. Alternative sources of MSCs are in high demand, especially from tissues that could be considered postsurgical wastes, for example, adipose-derived MSCs obtained after liposuction^35^ or S-MSCs gathered from plastic surgery.^20,36^ However, tumor-tropic properties of MSCs from different sources have yet to be understood, as only a few studies have investigated the migratory properties, such us motility and migration-related cell surface antigen markers, of MSCs isolated from more than one tissue.^37,38^

**Figure 3:**
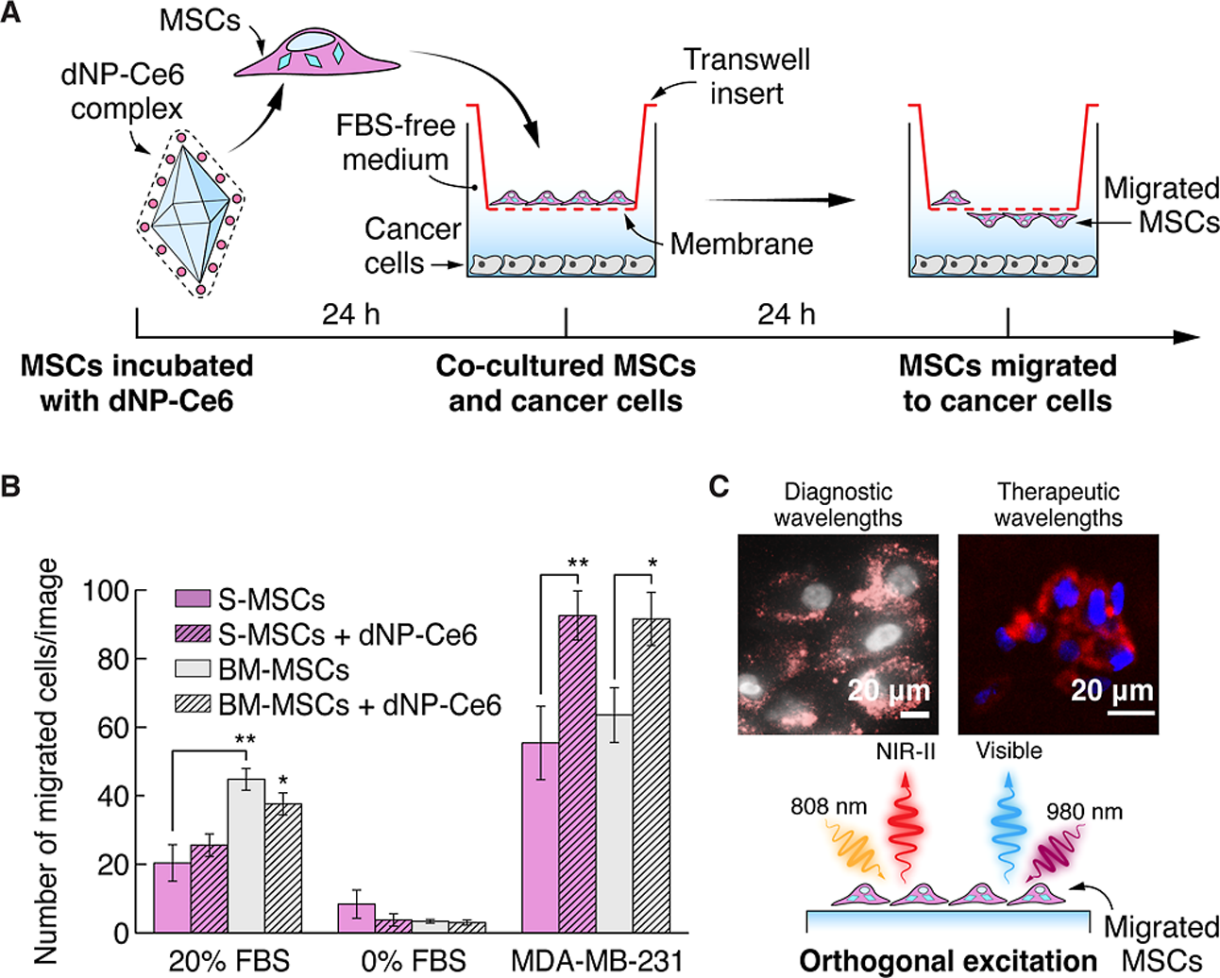
Migration studies performed using the Transwell migration assay. (A) Schematic illustration of the MSC migration experiment. (B) Number of migrated S-MSCs and BM-MSCs toward 0% FBS, 20% FBS, or MDA-MB-231 cells in the presence or absence of the dNP-Ce6 complex. Calculation for each group was performed from 5 independent images. *p ≤ 0.05; ** *p* ≤ 0.01. Error bars represent the standard deviation. (C) NIR-II and upconversion emission-based imaging of the dNP–Ce6 complex within MSCs after migration with different wavelength laser excitation. Blue/white – nuclei stained with Hoechst (λ_ex_ = 404 nm); red – dNP-Ce_6_ complex (λ_ex_ = 980 or 808 nm).

To fill this knowledge gap, we used a standardized Transwell migration assay (Figure 3A) to compare the migration capabilities of S-MSCs and BM-MSCs toward cancer cells with the dNP-Ce6 complex (0.1 mg/mL, 24 h of incubation) and without. MSCs were seeded into inserts with porous membrane, which were placed in cell culture plate wells filled with cell culture growth media without fetal bovine serum (0% FBS – no stimulus for migration, negative control), media complement with 20% FBS (chemoattractant stimulus, positive control), or MDA-MB-231 cells growing in FBS-free media (tumor-tropic stimulus). Note, we have previously demonstrated that S-MSCs do not migrate toward human mammary epithelial cell line MCF-10A,^36^ which frequently are used as healthy cells. Since the size of MSCs varies from 17 to 30 μm,^39^ MSCs have to reorganize themselves and squeeze through the 8 μm membrane pores, proving that they can migrate through obstacles similar to the ones encountered in vivo, e.g., blood capillary pores.^40^ Using this experimental design, we observed that S-MSCs and BM-MSCs were attracted more by the stimulus of MDA-MB-231 cancer cells than by 20% FBS (Figures 3B and S4). When comparing the migration properties of different MSCs, we observed that BM-MSCs migrate toward 20% FBS more efficiently than S-MSCs. In contrast, both types of MSCs migrated similarly toward MDA-MB-231 cancer cells. Intriguingly, we found that both S-MSCs and BM-MSCs migrated toward MDA-MB-231 cancer cells ∼1.5 times more effectively with the dNP-Ce6 complex than without (91–92 vs 55–64 cells/image) (Figure 3B; *p* < 0.05). Upon engulfment of dNP-Ce6 complex, MSCs can increase the S-phase fraction of their population and upregulate proteins associated with migratory capacity (c-Met, CCR1, and CXCR4 levels),^41^ thereby increasing their ability to systemically translocate to sites of inflammation. Enhanced tropism of MSCs to solid tumors and acute injury sites is linked with the overexpression of cMet, CCR1, and CXCR4 proteins^42−44^ and can be deliberately achieved by genetic modifications^42,45^ or engulfment of NPs.^41,43^

In tumor-tropic therapies, the nanotherapeutic cargo must retain its functionalities before, during, and after migration of the MSCs. Accordingly, we investigated the emission of the dNP-Ce6 complex in MSCs using 808 and 980 nm laser excitations after these cells migrated through the Transwell assay. Both excitation wavelengths are in the NIR region and allow us to switch between the decoupled functionalities of the dNPs: NIR-II imaging and upconversion-based PDT.^24^ As shown in Figure 3C, NIR emission was registered from inside the migrated cells under 808 nm excitation (detection range >850 nm, see Materials and Figure S6), and the excitation by 980 nm laser produced visible emission (detection range 620–755 nm). These results suggest that the intracellular dNP-Ce6 complex maintains its optical properties after the MSCs migrate through the 8 μm pores.

### 2.4 dNP-Ce6 Complex Penetration inside Tumor Tissue

**Figure 4:**
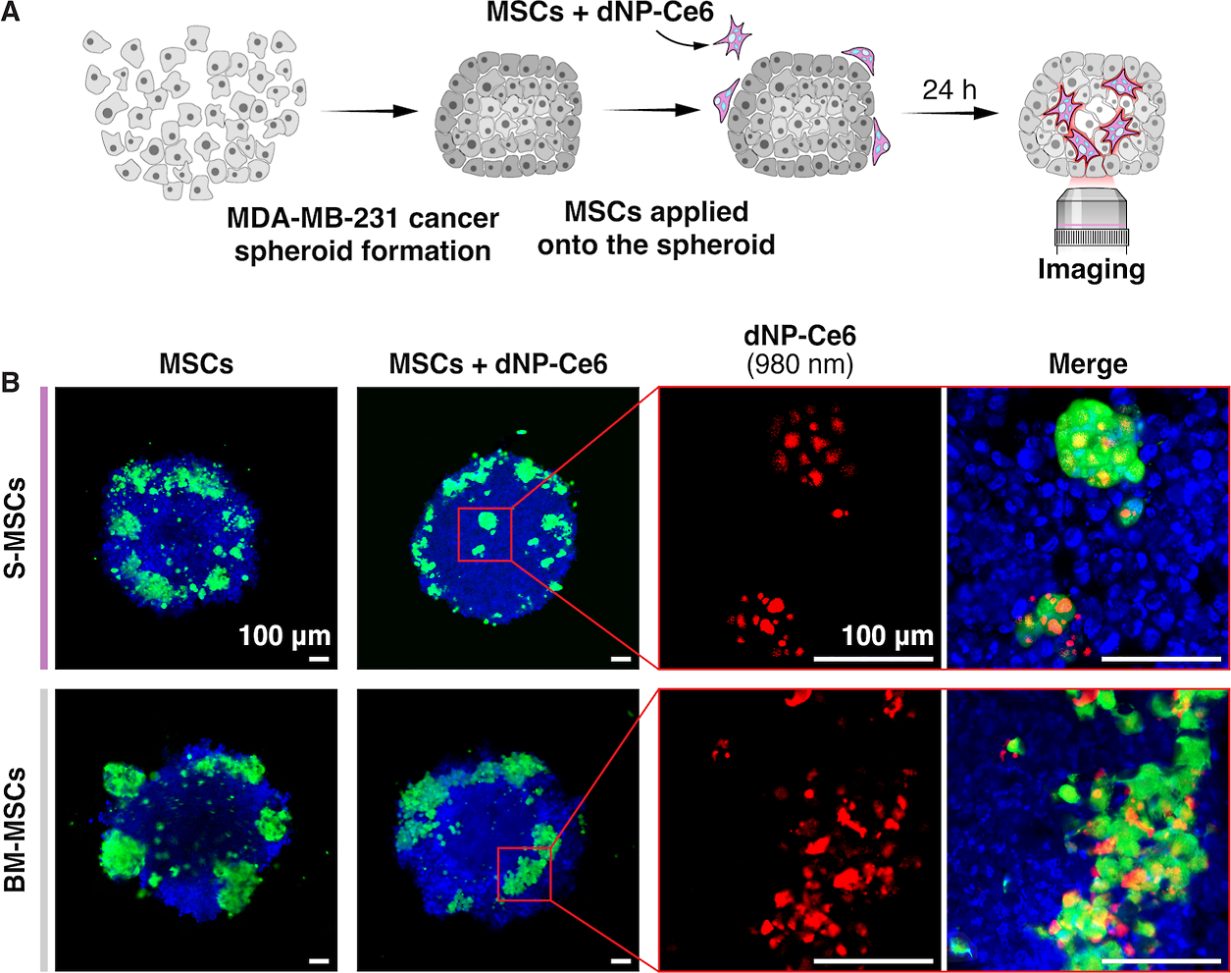
(A) Schematic illustration of the MSC penetration into the MDA-MB-231 spheroids experiment. (B) Fluorescence images of S-MSCs or BM-MSCs inside MDA-MB-231 spheroids without (leftmost images) or with the dNP-Ce_6_ complex (0.1 mg/mL). Blue – MDA-MB-231 nuclei stained with Hoechst (λ_ex_ = 404 nm), green – MSCs stained with calcein-AM (λ_ex_ = 488 nm); red – dNP-Ce6 complex (λ_ex_ = 980 nm). Scale bar −100 μm.

Motivated by the successful outcome of the migration studies, we examined the ability of MSCs to deliver the dNP-Ce6 complex inside MDA-MB-231 cellular spheroids, which was used as a representative model of a complex tumor.^10,24,46,47^ Following the formation of MDA-MB-231 spheroids, they were coincubated with calcein-AM-labeled MSCs. After 24 h, we observed that the MSCs had adhered, penetrated, and migrated inside the spheroids, with some cells reaching its center part (Figure 4). MSCs also interacted with each other to form small clusters inside the MDA-MB-231 spheroid. We hypothesize that these clusters are spheroid-like MSCs formations,^48^ possibly due to more significant MSCs-to-MSCs rather than MSCs-to-MDA-MB-231 interactions. MSCs express high levels of cell adhesion molecules (cadherins, integrins), which facilitate strong homophilic interactions,^49^ leading MSCs to adhere more readily to each other than to cancer cells. No significant difference between the migrations of S-MSCs and BM-MSCs was observed.

Since MSCs could migrate into deeper regions of MDA-MB-231 cellular spheroids, we investigated whether the MSCs maintain their tumor-penetration abilities while carrying the dNP-Ce6 complex. Loaded with the dNP-Ce6 complex, MSCs successfully penetrated the central parts of the MDA-MB-231 cellular spheroids, as indicated by calcein-AM and dNPs upconversion emissions (Figure 4). We did not detect any qualitative differences in the migration of the MSCs with and without the dNP-Ce6 complex.

Despite several studies on tumor-tropic properties of MSCs, only a few have investigated MSCs penetration into tumors.^47,50^ Earlier works have demonstrated that BM-MSCs can penetrate inside cellular spheroids and, in the case of Ferreira et al., can deliver cargo for NIR-induced photothermal therapy and chemotherapy.^47,50^ We corroborate these reports and show that S-MSCs could also be used for NPs delivery. Additionally, we for the first time demonstrated that inorganic rare earth-doped NPs could be used as MSCs cargos, as well.

### 2.5 Photodynamic Effect Evaluation

**Figure 5:**
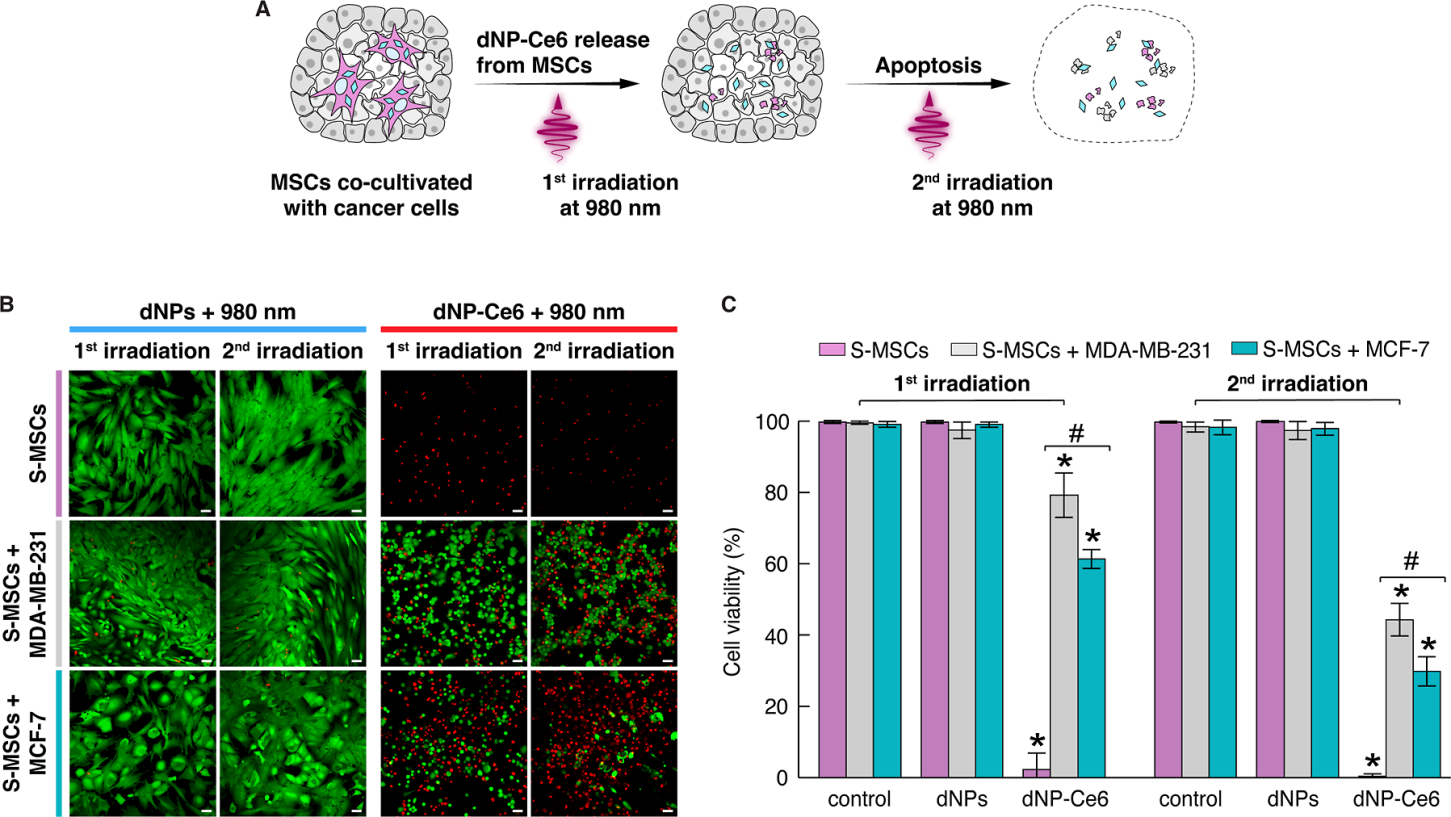
Two-dose photodynamic effect in monolayer and 3D cell cultures. (A) Schematic representation of the two-dose irradiation PDT. (B) Calcein-AM (green, live cells, λ_ex_ = 488 nm) and propidium iodide (red, dead cells, λ_ex_ = 543 nm) stained cells preincubated with dNPs or dNP-Ce_6_ complex after the first and second irradiation. The second irradiation was conducted 24 h after the first irradiation. Irradiation was performed with a 980 nm laser (0.9 W/cm^2^) for approximately 7 min, yielding an irradiation dose of 450 J/cm^2^. Scale bar – 50 μm. (C) Quantitative evaluation of the photodynamic effect. Mean (*n* = 3) is shown. Error bars indicate standard deviation. * Indicates a statistically significant difference between control and dNPs or dNP-Ce6 complex exposure (*p* < 0.05). # indicates a statistically significant difference between MDA-MB-231 and MCF-7 (*p* < 0.05).

Subsequently, we investigated the photodynamic activity of the dNP-Ce6 complex delivered to cancer cells by S-MSCs in 2D and 3D cell models. We used a two-dose 980 nm laser irradiation approach^20^ to ensure the release of the dNP-Ce6 complex into the medium after the first irradiation dose and damage to cancer cells after the second irradiation (Figure 5A).

We first examined the PDT effect using S-MSCs and cancer cell co-cultures grown in monolayers. S-MSCs were incubated with the dNP-Ce6 complex and then co-cultured with cancer cells for 24 h; at this point, the two-dose 980 nm laser irradiation was applied. The first irradiation dose affected only S-MSCs because only these cells have accumulated the dNP-Ce6 complex (Figure 5B). Damaged S-MSCs released the dNP-Ce6 complex into the surrounding microenvironment to be taken up by cancer cells (during 24 h interval between irradiations). Subsequently, we irradiated the cells for the second time, with the result that most cancer cells died or showed apoptosis-related damage, such as shrinkage and fragmentation into membrane-bound bodies. To assess the PDT effect quantitatively, we estimated the cell viability from the fluorescence images taken after each dose of laser irradiation. The first irradiation dose led to a complete eradication of S-MSCs incubated with the dNP-Ce6 complex (0% viability), whereas cell viability in samples co-cultured with MDA-MB-231 and MCF-7 cells remained at 79 and 61%, respectively (Figure 5C, first irradiation). However, the second irradiation further decreased cancer cell viability, i.e., cell viability in samples co-cultured with MDA-MB-231 and MCF-7 cells dropped to 44 and 30%, respectively (Figure 5C, second irradiation). In addition, a similar two-dose irradiation of cell cocultures with Ce6-free dNPs showed no significant reduction in cell viability. To rule out photothermal damage to cells, we performed a control experiment by irradiating the cells with a 980 nm laser for up to 7 min at 0.9 W/cm^2^ laser power density (as in the above-described experiments); we observed no damage to cells in this case (Figure S5).

**Figure 6:**
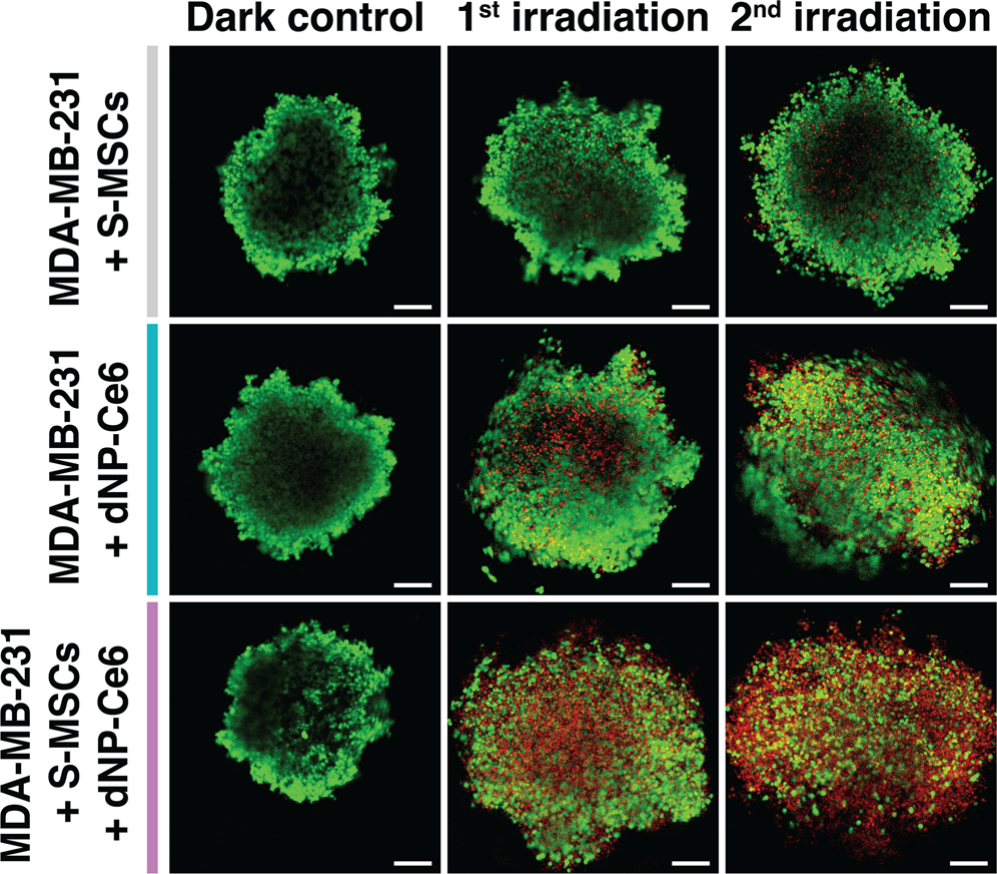
PDT effect on MDA-MB-231 cellular spheroids. MDA-MB-231 cellular spheroids were incubated with S-MSCs, or dNP-Ce6 complex (0.1 mg/mL), or S-MSCs carrying the dNP-Ce_6_ complex (0.1 mg/mL). After 24 h of incubation, two-dose irradiation was applied (with 24 h break). Spheroids were stained with fluorescent viability dyes: green – calcein-AM stained (live) cells (λ_ex_ = 488 nm); red – propidium iodide stained (dead) cells (λ_ex_ = 543 nm). Scale – 200 μm.

Finally, we investigated the suitability of the dNP-Ce6 complex for stem-cell-based PDT using cellular cancer spheroids as a tumor model. As before, S-MSCs were incubated with the dNP-Ce6 complex and then incubated with the fully formed MDA-MB-231 cellular spheroids for 24 h. Then, two-dose irradiation was performed, maintaining the same experiment design as used for cell monolayers. Irradiation with a 980 nm laser led to a decrease in the viability of cells and the disintegration of spheroids in the case when S-MSCs carried a dNP-Ce6 complex (Figure 6). A notably higher effect was observed in spheroids where the dNP-Ce6 complex was carried by S-MSCs, compared to passive accumulation of the dNP-Ce6 complex. In control spheroids, where S-MSCs were applied without the dNP-Ce6 complex, the two-dose irradiation of cells with a 980 nm laser had no significant effect – cells remained viable, and spheroids maintained their integrity. We thus observed that S-MSCs improved the delivery of the dNP-Ce6 complex into cancer spheroids leading to an enhanced PDT effect and damage to MDA-MB-231 cancer cells under the therapeutic 980 nm irradiation. Overall, both 2D and 3D in vitro model systems have proved that the dNP-Ce6 complex can be transported by S-MSCs to tumors and cause damage to cancer cells via two-dose NIR-activated PDT. Additionally, the use of MSCs for transporting photoactive nanotherapeutics can be regarded as biocompatible because these carrier cells are also eradicated during laser irradiation.

## 3 Conclusions

We investigated two types of MSCs (extracted from skin – S-MSCs–and from bone marrow – BM-MSCs) as carriers of a theranostic complex of rare earth-doped nanoparticles and photosensitizer Ce6 (dNP-Ce6). Specifically, we benchmarked the tumor-tropic properties of S-MSCs, which are patient-friendly to isolate, against the most researched and well-known BM-MSCs. Both S-MSCs and BM-MSCs exhibited effective penetration and internalization into cancer cell spheroids when loaded with the dNP-Ce6 complex, with an observed enhanced migration toward cancer cells compared to their nonloaded counterparts. Significantly, we demonstrated that the tumor-homing properties and capabilities for transferring NPs to cancer cells do not depend on the tissue from which MSCs were isolated. Although further research is still necessary, these findings indicate that various MSCs are suitable for stem-cell-based drug delivery. After successfully delivering the dNP-Ce6 complex via MSCs, we employed a two-dose NIR irradiation strategy to release the dNP-Ce6 complex from the MSCs and maximize damage to the tumor cells. Our results demonstrated that active dNP-Ce6 complex transportation with MSCs leads to an increased PDT effect in cancer cell spheroids, compared to passive delivery of the dNP-Ce6 complex. Thus, using MSCs to directly deliver therapeutic NPs to tumors could significantly minimize damage to healthy tissues and reduce side effects.

Overall, we showed that in combination with suitably designed NPs that display decoupled therapeutic and imaging abilities, the unique approaches of MSCs-based drug delivery hold great promise for advancing cancer therapy research.

## 4 Experimental Section

### 4.1 Synthesis

#### 4.1.1 Materials

Lu_2_O_3_ (REacton, 99.9%), Er_2_O_3_ (REacton, 99.999%), Nd_2_O_3_ (REacton, 99.999%), Yb_2_O_3_ (REacton, 99.99+%) trifluoroacetic acid (99%), 1-octadecene (ODE, 90%), oleic acid (OA, 90%), trisodium citrate (99%), and lithium trifluoroacetate (97%) were purchased from Alfa Aesar (USA). Oleylamine (OM, 70%), bis(hexamethylene)triamine (BHMT, 95%), and poly(maleic anhydride-*alt*-1-octadecene) (PMAO, average Mn = 30,000–50,000) were obtained from Sigma-Aldrich. The amphiphilic chlorin e6 (Ce6) photosensitizer was purchased from Frontier Scientific Inc., USA. All chemicals were used without further purification.

#### 4.1.2 Preparation of Precursors for dNP Synthesis

In a typical experiment, to prepare LiLuF_4_:Nd^3+^ rare earth nanoparticle cores, stoichiometric amounts of Nd_2_O_3_ (0.0625 mmol) and Lu_2_O_3_ (2.4375 mmol) were first mixed with 5 mL of trifluoroacetic acid and 6 mL of water in a 50 mL three-neck round-bottom flask, and the mixture was allowed to reflux under vigorous magnetic stirring at 85 °C until clear. The temperature of the solution was then reduced to 60 °C, the stirring was stopped, and the residual trifluoroacetic acid and water were evaporated. Precursors for the LiLuF_4_ and LiYbF_4_:Er^3+^ shells were prepared following a similar procedure, using Lu_2_O_3_ and Yb_2_O_3_ and Er_2_O_3_.

#### 4.1.3 Synthesis of dNPs

Preparation of core NPs for the dNPs was carried out via a modified two-step thermal decomposition method following first nuclei (FN) synthesis, which were further used as seeds to form the desired cores.

#### 4.1.4 FN Formation

FN were formed via a hot-injection approach. Solution A was prepared by mixing 7 mL each of OA and OM, and 14 mL of ODE in a 100 mL three-neck round-bottom flask. Besides, to prepare Solution B, 2.5 mmol of lithium trifluoroacetate, 3 mL of OA, 3 mL of OM, and 6 mL of ODE were added to a 50 mL three-neck round-bottom flask containing the dried rare earth trifluoroacetate precursors for the core. The OM was added to Solution B after the precursors were dissolved under vacuum in a pure OA/ODE mixture. The temperature of both Solutions A and B was increased to 125 °C and they were kept under vacuum with vigorous stirring for 30 min to degas the mixtures. After complete degassing, the temperature of Solution A was increased to 330 °C under an Ar atmosphere. Solution B was then injected into Solution A by using a pump-syringe system (Harvard Apparatus Pump 11 Elite) at a 1.5 mL/min injection rate. The temperature of the reaction solution was maintained at 330 °C for 1 h. Afterward, the solution was allowed to cool to room temperature (heating mantle was removed), maintaining magnetic stirring and an Ar atmosphere. The resulting solution is termed the stock solution in the next section.

#### 4.1.5 Core Synthesis following Stabilization

Core NPs were prepared following stabilization of FN with an excess amount of OA. In a typical procedure, FN (1.5 mmol, ∼21 mL of stock solution) were mixed with 15 mL each of OA and ODE in a 100 mL three-neck round-bottom flask. The temperature of the solution was raised to 125 °C and the solution was kept for 30 min under vacuum and vigorous magnetic stirring. After degassing, the temperature of the solution was further increased to 330 °C under an Ar atmosphere and reaction was continued for 1 h. Afterward, the solution was allowed to cool down to room temperature (heating mantle was removed), maintaining the magnetic stirring and Ar atmosphere. A small portion (∼0.5 mL) of core NPs was sampled for TEM characterization.

#### 4.1.6 Shelling of the Cores

Core/shell/shell/shell dNPs were successfully synthesized following the epitaxial growth of different functional shells on the core NPs via the hot-injection approach. To grow the blocking LiLuF_4_ shell, 1.1 mmol (∼37 mL) of cores were placed in a 100 mL three-neck round-bottom flask. 1.5 mL of OA and 1.5 mL of ODE were added to the same flask to make a total volume of 40 mL (Solution A). The LiLuF_4_ shelling mixture was prepared as Solution B: ∼1.5 mmol of lithium trifluoroacetate together with 5 mL of each OA and ODE were added to a 50 mL three-neck round-bottom flask containing ∼1.5 mmol of lutetium trifluoroacetate shelling precursors. The temperatures of both Solutions A and B were raised to 125 °C and they were degassed under a vacuum with vigorous stirring for 30 min. Solution A was then heated to 320 °C under an Ar atmosphere. Subsequently, Solution B was injected into Solution A using a pump-syringe system at a 0.5 mL/min injection rate. The temperature of the reaction solution was maintained at 320 °C for 1 h. In parallel, the LiYbF_4_:Er^3+^ shelling mixture (Solution C) was prepared from lithium (1.1 mmol), ytterbium (1.089 mmol), and erbium (0.011 mmol) trifluoroacetates and 5 mL each of OA and ODE as described above. Before injection of Solution C, 4.55 mL of the reaction mixture was aliquoted for sampling. Solution C was injected at a 0.5 mL/min injection rate and was left to react for 1 h. In parallel, the LiLuF4 shelling mixture (Solution D) was prepared from lithium (12.87 mmol) and lutetium (12.87 mmol) trifluoroacetates and 10 mL each of OA and ODE as described above. Before injection of Solution D, 5.55 mL of the reaction was aliquoted for sampling. Solution D was injected at a 0.5 mL/min injection rate in two 10 mL steps with a 1 h reaction time after each step. After the reaction was complete, the solution was allowed to cool to room temperature (heating mantle was removed), maintaining the magnetic stirring and Ar atmosphere. The resultant core/shell/shell/shell dNPs were precipitated with ethanol and collected via centrifugation at a 5400 RCF for 15 min. The precipitate was then washed with a hexane/ethanol mixture (1/4 v/v) and recollected via centrifugation at 5400 RCF for 15 min. This process was repeated two more times. Finally, the oleate-capped dNPs were redispersed in hexane for further structural and optical characterization and for subsequent transfer to water.

#### 4.1.7 dNPs Transfer to Water and dNP-Ce6 Complex Formation

Oleate-capped dNPs were transferred to water following literature-reported amphiphilic polymer coating.^30^ Chloroform dispersion of dNPs (10 mg) was mixed with 3 mL of chloroform and 1 mL of PMAO (stock: 1.4 g in 20 mL chloroform) under stirring for 2 h. After the addition of a BHMT (0.4 mg, 100 μL) cross-linker, the mixture was sonicated for 30 min. The solvent was evaporated under Ar flow and magnetic stirring on a warm hot plate (∼60 °C), and to remove any residual chloroform, the resultant transparent film was left to dry in air on a hot plate overnight. Then, the film was redispersed in 20 mL of 0.02 M aqueous NaOH solution under sonication until it was completely clear (∼1 h). The dispersion was subjected to filtration with a disk-type 0.45 μm pore-sized filter. The resulting PMAO-cross-linked dNPs were isolated by centrifugation (9500 RCF, 45 min) and redispersed in distilled water. The dNP-Ce6 complex was prepared in an analogous way, except that the redispersion of dNPs film was done with 17.6 mL of 0.02 M NaOH and 2.4 mL of Ce6 (0.84 μM stock solution in 0.02 M NaOH).

### 4.2 Characterization

#### 4.2.1 Structural Characterization

The crystallinity of dNPs was determined via powder X-ray diffraction (XRD) analysis on a Bruker D8 Advance Diffractometer using Cu Kα radiation (λ = 1.5418 Å). Morphology and the size distribution analysis were carried out using a Philips Tecnai 12 (USA) transmission electron microscope operated at an accelerating voltage of 80 kV. Samples were supported on carbon-coated copper grids. The nanoparticle size was determined from TEM images using ImageJ image analysis software with a set size of at least 150 individual particles per sample.

#### 4.2.2 Optical Characterization

Emission spectra of the dNPs were acquired using 793 nm (Changchun New Industries Optoelectronics Tech. Co., China) or 960 nm (BWT, China) laser diode excitation. The upconversion emission was recorded with an AvaSpec-ULS2048L spectrometer (Avantes, The Netherlands). Stray light from the excitation source was removed with a short-pass 825 nm filter (Newport corp., USA). The downshifting emission of dNPs in the near-infrared region was collected with a Shamrock 500i monochromator (Andor, Ireland) equipped with an iDus InGaAs 1.7 NIR detector (Andor, Ireland). In order to remove any stray light from the excitation source, a long-pass 830 (or 980 nm) filter (Semrock, USA) was used.

#### 4.2.3 Cell Lines and Cell Culturing

Primary S-MSCs were isolated from human skin samples that were obtained from redundant eyelid skin left during blepharoplasty in the Baltic – American Medical & Surgical Clinic. S-MSCs were isolated as described earlier by using a modified explant culture method.^20^ BM-MSCs were isolated from healthy human bone marrow samples, which were received from Vilnius University Hospital Santaros Klinikos after joint surgery as described earlier.^51^ S-MSCs and BM-MSCs were used in accordance with authorized approval from the Vilnius Regional Biomedical Research Ethics Committee no. 158200–18/6–1036–548 and no. 158200–14–741–257. Donors were informed and signed an informed consent form. S-MSCs and BM-MSCs were grown in Dulbecco’s Modified Eagle Medium (DMEM) with GlutaMAX supplement and containing F-12 nutrient mixture (DMEM/F-12, 3:1 v/v), 10% fetal bovine serum (FBS), 100 U/mL penicillin, and 100 μg/mL streptomycin. All reagents were from Gibco, Thermo Fisher Scientific, United Kingdom (UK). MSCs used in the study were between 2–8 passage.

The luminal A human breast cancer cell line MCF-7 was purchased from the European Collection of Authenticated Cell Cultures (ECACC, UK) and aggressive triple-negative human breast cancer cell line MDA-MB-231 was purchased from the American Cell Culture Collection (ATCC, USA). Cancer cells were cultured in DMEM supplemented with 10% FBS, 100 U/mL penicillin, and 100 μg/mL streptomycin.

All cells were grown in a humidified chamber (Binder, Germany) at 37 °C with 5% CO_2_. Cells were cultured in 25 or 75 cm^2^ culture flasks with a filter (Thermo Fisher Scientific, USA) and passaged 2 times a week.

#### 4.2.4 MSC Characterization

S-MSCs and BM-MSCs were characterized according to the criteria of the International Society of Cellular Therapy.^52^ Immunophenotype detection was performed by staining 1 × 10^5^ cells with the following antibodies conjugated with the appropriate fluorochromes: CD90-fluorescein isothiocyanate (FITC) (BD Biosciences, USA), CD73-FITC, CD105-phycoerythrin (PE), CD45-FITC, CD34-allophycocyanin (APC), and CD14-PE (all five from eBioscience, Thermo Fisher Scientific, USA). Additionally, cells were stained with CD44-FITC (Thermo Fisher Scientific, USA). The stained and washed cells were analyzed using a BD LSR II (BD Biosciences) flow cytometer by collecting 10,000 viable cells.

MSCs were seeded into 8-chambered cover glass plates (Nunc, Lab-Tek) at 4 × 10^3^ cells/chamber and 8 × 10^3^ cells/chamber for osteogenesis and adipogenesis differentiations, respectively. After 96 h, the cells were subjected to an osteogenic or adipogenic medium (Gibco, StemPro). For chondrogenic differentiation, 2 × 10^5^ MSCs were suspended in chondrogenic differentiation medium (Gibco, StemPro) and centrifuged at 500*g* for 5 min in 15 mL polypropylene centrifuge tubes (nerbe plus GmbH, Germany). Cells of the pellet culture were grown with loosened caps to permit gas exchange. All of the cells were cultivated with differentiation medium for 21 days with medium exchange every 3–4 days. Afterward, osteogenic and adipogenic samples were fixed with 10% neutral buffered formalin for 40 min and treated with 1% (w/v) Alizarin Red S (Sigma-Aldrich, Germany) dissolved in deionized water or 0.5% (w/v) Oil Red O (Sigma-Aldrich, Germany) dissolved in isopropanol, respectively, for 30 min. The formed pellet in chondrogenic medium was cut into 15 μm thick slices with a cryomicrotome and stained for 30 min with 1% (w/v) Toluidine Blue (Sigma-Aldrich, Germany) in deionized water. MSC characterization results are provided in Supporting Information Figure S3.

#### 4.2.5 Intracellular Localization of the dNP-Ce6 Complex

For the intracellular localization studies, cells were seeded in 8-well chamber slides (Lab-Tek II, Nunc, Thermo Fisher Scientific, USA), S-MSCs, and BM-MSCs at a density of 7 × 10^3^ cells/well and MCF-7 or MDA-MB-231 at a density of 3 × 10^4^ cells/well. After 24 h, the cell growth medium was replaced with a new medium containing the 0.1 mg/mL dNP-Ce6 complex and incubated for 24 h under standard conditions (37 °C, 5% CO_2_). The cells were then washed 3 times with warm Dulbecco’s phosphate-buffered saline (DPBS) (Cegrogen Biotech, Germany) and stained with 20 μg/mL Hoechst 33342 (Sigma-Aldrich, USA) for 10 min to label nuclei. Then, the cells were washed with DPBS, fixed with 4% paraformaldehyde (Sigma-Aldrich, Germany) for 15 min, permeabilized with 0.2% Triton X-100 (Sigma-Aldrich, USA) for 3 min, and stained with 165 mM Alexa Fluor 488 Phalloidin (Thermo Fisher Scientific, UK) for 20 min to label F-actin filaments. Eventually, the cover glass was mounted with Cytoseal 60 mounting medium (Thermo Fisher Scientific, UK).

Intracellular accumulation of the dNP-Ce6 complex in cells was evaluated with a Nikon Eclipse Te2000–S C1si laser scanning confocal microscope (Nikon, Japan) using a 60× NA 1.4 oil immersion objective (Plan Apo, Nikon, Japan). A 404 nm diode laser (Melles Griot, USA) was used to excite Hoechst 33342 and Ce_6_, a 488 nm Ar laser (Melles Griot, USA) for Alexa Fluor 488 Phalloidin and a 980 nm laser (Changchun New Industries Optoelectronics Tech. Co., China) for the dNP-Ce6 complex. Image processing was performed using EZ-C1 v3.90 software (Nikon, Japan) and ImageJ 1.48 software (National Institute of Health, USA).

#### 4.2.6 Cell Viability Assay

The dark toxicity of dNPs and the dNP-Ce6 complex in S-MSCs, BM-MSCs, MCF-7, and MDA-MB-231 was evaluated using the lactate dehydrogenase (LDH) cytotoxicity assay (Thermo Fisher Scientific, USA). Cells were seeded in 96-well plates (BD Falcon, USA) at a density of 5 × 10^3^ MSCs/well and MCF-7 or MDA-MB-231 at a density of 1.5 × 10^4^ cells/well. After 24 h, cells were treated with four different concentrations of dNPs and the dNP-Ce6 complex (0.05; 0.1; 0.2; 0.3 mg/mL) diluted in cell growth medium, and 150 μL of the final solution was added to each well. Untreated cells were used as control groups. Plates were incubated in the dark at 37 °C and 5% CO_2_ for 24 h. After incubation, the LDH assay was performed according to the manufacturer’s protocol. Finally, cytotoxicity (%) was converted to cell viability (100%—cytotoxicity (%)) for better visualization.

#### 4.2.7 Transwell Migration Assay

To verify the tumor-tropic ability of BM-MSCs and S-MSCs, cell migration assays were performed using Transwells with 0.8 μm pore polycarbonate membrane inserts (Corning, USA). The assay was performed according to the manufacturer’s recommendations with three different chemoattractants: MSC growth medium supplemented with 20% FBS (positive control), MSC serum-free medium (negative control), and MDA-MB-231 cancer cells.

Briefly, MDA-MB-231 cells were seeded at a density of 5 × 10^4^ cells/well in a 24-well plate. The next day, the cell growth medium was replaced with serum-free medium and kept until the end of the experiment (for 3 days). On the same day as the cancer cells, the MSCs were also seeded at a density of 3 × 10^4^ cells/well in a full growth medium. Next day, the medium of MSCs was replaced with a fresh medium (for the control cells) or a medium containing the dNP-Ce6 complex (0.1 mg/mL). After 24 h of incubation, control cells and MSCs treated with the dNP-Ce6 complex were collected via trypsinization, resuspended in 100 μL of serum-free MSC medium and plated on inserts (3 × 10^4^ cells/insert) for 24 h of migration under standard culturing conditions in a humidified incubator with 5% CO_2_ and 37 °C. The seeding procedure for migration of MSCs toward other chemoattractants (20% of FBS and serum-free medium) was identical to that for inserts for MDA-MB-231 cells.

Nonmigrated cells were wiped out from the upper side of the inset with a cotton swab soaked in sterile distilled water. Migrated cells were fixed with 4% paraformaldehyde for 15 min and stained with 25 μg/mL Hoechst for 15 min. Migrated cells were visualized under a Nikon Eclipse Te2000–S C1si laser scanning confocal microscope (Nikon, Japan) with a 20× objective. Number of migrated cells per image was calculated from five different microscope fields. Results are presented as the average ± SD (standard deviation).

#### 4.2.8 NIR Imaging under 808 nm Excitation

Near infrared luminescence images were acquired on a modified commercial inverted microscope (Eclipse Ti2–U, Nikon). Visible luminescence images were collected first using a scientific complementary metal oxide semiconductor (sCMOS) camera (Orca-Flash4.0, Hamamatsu), a 50× NA 0.80 objective (LU Plan Fluor, Nikon), a light emitting diode (LED) lamp (pE-300light-series, CoolLED) as the excitation source, and a DAPI filter cube (Ex. 352–402 nm, DC409, Em. 417–477 nm). NIR images were acquired using an InGaAs camera (C-RED 2, First Light Imaging Corp.), a 50× NA 0.65 objective optimized for NIR (LCPlan N, Olympus), an 808 nm single-mode diode laser (BTF14, Lumics) as excitation source, and a NIR filter cube mounting the following filters: SP850, DMLP900, and LP850. The power density at the stage was approximately 167 W/cm^2^. The images were analyzed with ImageJ software.

#### 4.2.9 Cellular Spheroids

Cellular spheroids were formed from MDA-MB-231 cells using Nunclon Sphera 96 U-Shaped Bottom plates (Thermo Fisher Scientific, Japan). MDA-MB-231 cells were seeded at a density of 1.5 × 10^4^ cells/well in a standard cell culture medium and centrifuged with LMC-3000 (BioSan, Latvia) at 120 RCF for 6 min before incubation at 37 °C, 5% CO_2_. MDA-MB-231 spheroids formed within 4 days when cells are grown under standard conditions without changing the media. The nuclei of MDA-MB-231 spheroids were stained with 20 μg/mL Hoechst 33342 for 24 h and washed with DPBS. MSCs were preincubated with 0.1 mg/mL dNP-Ce6 complex in 8.8 cm^2^ Petri dishes (Greiner bio-one, Germany) for 24 h, washed 3 times with DPBS, and stained with 2 μM calcein-AM (Thermo Fisher Scientific, USA) for 30 min. After harvesting, MSCs (1500 cells/well), uploaded with the dNP-Ce6 complex, were placed and incubated with fully formed spheroids of MDA-MB-231 cells for 24 h. Control wells were incubated with MSCs without the dNP-Ce6 complex. The ability of MSCs to extravasate the spheroids was evaluated with a Nikon Eclipse Te2000–S C1si laser scanning confocal microscope at 10× and 60× magnification.

#### 4.2.10 Singlet Oxygen Generation

Singlet oxygen indicator SOSG (Singlet Oxygen Sensor Green; Invitrogen, USA) was used to study the singlet oxygen generation. The SOSG reagent was dissolved in methanol to 5 mM and diluted in distilled water to a 1 μM working concentration. Four different samples were prepared for the evaluation of singlet oxygen generation: dNP-Ce6 complex and SOSG, dNPs and SOSG, Ce6 and SOSG, and SOSG only. The samples were irradiated for 30 min with a 980 nm CW laser (P_980_ = 1.55 W). Magnetic stirring was used throughout the irradiation experiment, and temperature was kept constant. The control samples were kept in the dark. SOSG fluorescence spectra (515–600 nm) were measured at specific time intervals using an Edinburgh Instruments FLS920 fluorescence spectrometer (Edinburgh Instruments Inc., UK). SOSG fluorescence was measured after each dose of irradiation.

#### 4.2.11 Photodynamic Therapy In Vitro

MDA-MB-231 and MCF-7 cells were seeded in eight-chamber coverglass plates (Lab-Tek, Thermo Fisher Scientific, USA) at a density of 1.5 × 10^4^ cells/well and S-MSCs in 8.8 cm^2^ Petri dishes. After attachment, S-MSCs were incubated with 0.1 mg/mL dNPs or dNP-Ce6 complex for 24 h, washed with DPBS, trypsinized, and then 7 × 10^3^ cells/well added to the 8-chamber coverglass plate with MDA-MB-231 or MCF-7 cells. S-MSCs and cancer cells were cocultured for up to 24 h. Irradiation was performed in two doses with a 980 nm laser (P_980_ = 0.9 W) to achieve a total dose of 450 J/cm^2^. There was a 24 h break between irradiations. After each irradiation, cells were stained with viability/cytotoxicity dyes, i.e., 2 μM calcein-AM (green fluorescence, stains viable cells) and 4 μM propidium iodide (red fluorescence, stains nonviable cells) (ROTH, Germany) and examined under a Nikon Eclipse Te2000–S C1si laser scanning confocal microscope using a 20*x*/0.5 NA objective (Nikon, Japan). The photodynamic effect was evaluated by counting viable and nonviable cells using ImageJ software. Results are presented as average ± SD (standard deviation).

To demonstrate the photodynamic effect in a three-dimensional cell culture, MDA-MB-231 cells were seeded in Nunclon Sphera 96 U-Shaped-Bottom plates at a density of 1.5 × 10^4^ cells/well and centrifuged at 120 RCF for 6 min before being incubated at 37 °C, 5% CO_2_. The formed MDA-MB-231 spheroids were incubated with the dNP-Ce6 complex (0.1 mg/mL) or with S-MSCs containing the dNP-Ce6 complex (1.5 × 10^3^ cells/well). S-MSCs were preincubated with 0.1 mg/mL dNP-Ce6 complex in 8.8 cm^2^ Petri dishes for 24 h, washed with DPBS, harvested, and incubated with MDA-MB-231 cell spheroids for 24 h. Control wells were incubated with S-MSCs (1.5 × 10^3^ cells/well) without the dNP-Ce6 complex. Next, irradiation and staining were performed as described above with the cell monolayer. Spheroids were imaged by confocal microscopy at 10× magnification.

#### 4.2.12 Statistical Analysis

All experiments were repeated at least 3 times. The results include the mean and standard deviations of independent experiments. Statistical significance was assessed using the two-tailed Student’s *t*-test. Differences were considered statistically significant at *p* ≤ 0.05.

## Supporting Information Available

Additional information, including characterization of dNPs, characterization of MSCs, confocal microscope images of MSCs nuclei after Transwell migration assay, photothermal effect on S-MSCs, MDA-MB-231 and MCF-7 cell lines, and NIR imaging under 808 nm excitation (PDF)
