# Supplementary material for: Synergistic Enhancement of Photodynamic Cancer Therapy with Mesenchymal Stem Cells and Theranostic Nanoparticles
Source: ACS Appl Mater Interfaces. 2024 Sep 10;16(37):49092–103. doi: 10.1021/acsami.4c10098 (PMC11420871; doi:10.1021/acsami.4c10098)
Supplement: Supplementary file 1 — am4c10098_si_001.pdf [file am4c10098_si_001.pdf]

SUPPORTING INFORMATION

Synergistic Enhancement of Photodynamic Cancer Therapy with Mesenchymal Stem Cells and Theranostic Nanoparticles

Greta Butkiene[1], Aleja Marija Daugelaite[1,2], Vilius Poderys[1], Riccardo Marin[3,4], Simona Steponkiene[1], Evelina Kazlauske[1,5], Ilona Uzieliene[6], Dainius Daunoravicius[7], Daniel Jaque[3,4,8], Ricardas Rotomskis[1,9], Artiom Skripka[3,10*], Fiorenzo Vetrone[10,11*], Vitalijus Karabanovas[1,5*]

[1]Biomedical Physics Laboratory of the National Cancer Institute, P. Baublio St. 3b, Vilnius LT-08406, Lithuania

[2]Faculty of Medicine, Vilnius University, M. K. Ciurlionio g. 21, Vilnius LT-03101, Lithuania

[3]Nanomaterials for Bioimaging Group (nanoBIG), Departamento de Física de Materiales, Facultad de Ciencias, Universidad Autónoma de Madrid, Madrid 28049, Spain

[4]Institute for Advanced Research in Chemical Sciences (IAdChem), Universidad Autónoma de Madrid, Madrid 28049, Spain

[5]Department of Chemistry and Bioengineering, Vilnius Gediminas Technical University, Sauletekio Ave. 11, Vilnius LT-10223, Lithuania

[6]Department of Regenerative Medicine, State Research Institute Centre for Innovative Medicine, Santariskiu g. 5, Vilnius LT-08406, Lithuania

[7]Clinicus Vilnius, V. Grybo g. 17-135, Vilnius LT-10318, Lithuania

[8]Nanomaterials for Bioimaging Group (nanoBIG), Instituto Ramón y Cajal de Investigación Sanitaria (IRYCIS), Hospital Ramón y Cajal, Madrid 28034, Spain

[9]Biophotonics Group, Laser Research Center, Physics Faculty, Vilnius University, Sauletekio Ave. 9, Vilnius LT-10222, Lithuania

[10]Centre Énergie, Matériaux et Télécommunications, Institut National de la Recherche Scientifique (INRS), Université du Québec, Varennes, Québec J3X 1P7, Canada

[11]Centre Québécois sur les Matériaux Fonctionnels (CQMF)/Québec Centre for Advanced Materials (QCAM), Montréal, Québec J3X 1P7, Canada

Present address: Department of Chemistry, Oregon State University, Corvallis, Oregon 97331, United States (A.S.)

*Corresponding authors: artiom.skripka@oregonstate.edu, fiorenzo.vetrone@inrs.ca, and vitalijus.karabanovas@nvi.lt

Table of Contents

Supporting results 3

Characterization of dNPs 3
Characterization of MSCs 3
Migration study using Transwell migration assay 5
Photothermal effect 6
NIR imaging under 808 nm excitation 7

References 8

Supporting results

Characterization of dNPs

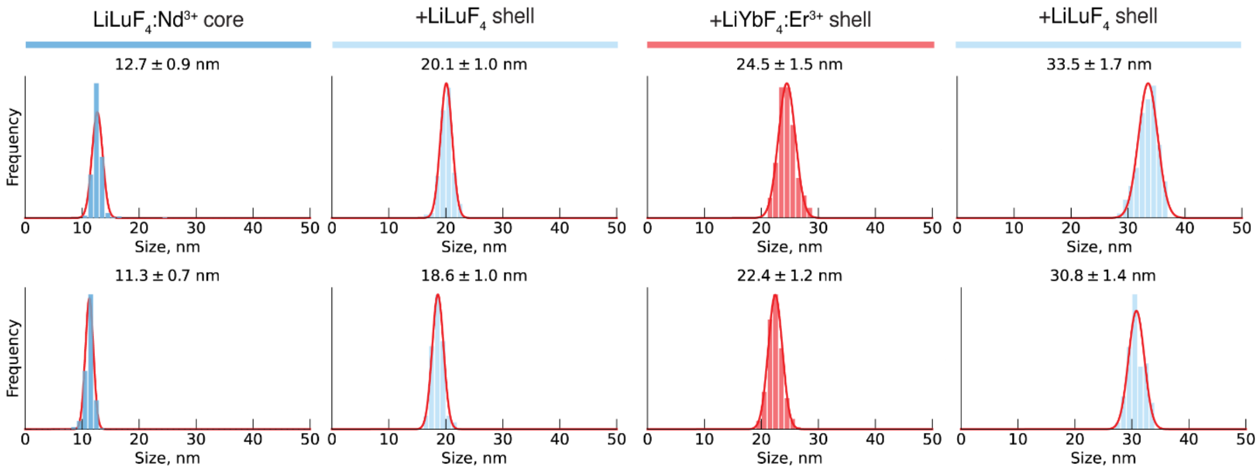

Figure S1. Size distribution histograms (major axis – top, minor – bottom) of core/shell/shell/shell $LiLuF_4:Nd^{3+}/LiLuF_4/LiYbF_4:Er^{3+}/LiLuF_4$ dNPs, together with average size and one standard size deviation, following each synthesis step.

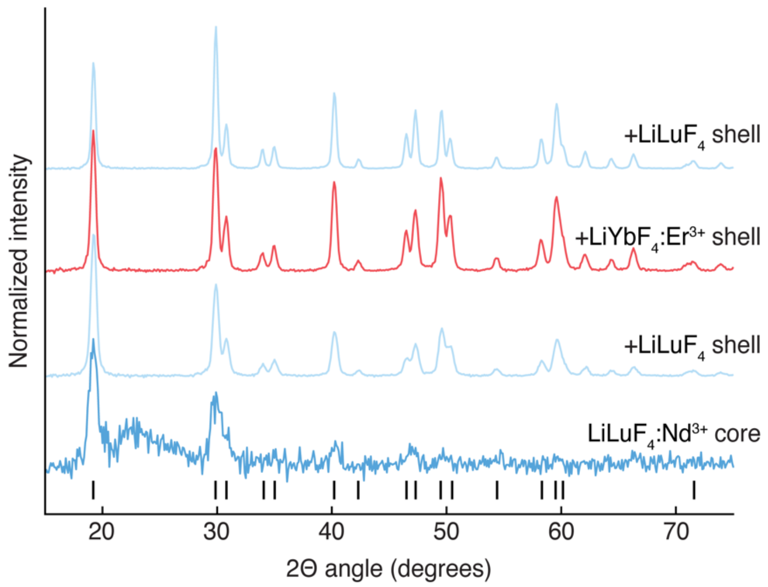

Figure S2. XRD patterns of core/shell/shell/shell $LiLuF_4:Nd^{3+}/LiLuF_4/LiYbF_4:Er^{3+}/LiLuF_4$ dNPs following each synthesis step. Reference $LiLuF_4$ pattern (PDF# 00-027-1251) is shown as black marks.

Characterization of MSCs

S-MSCs and BM-MSCs were confirmed to meet criteria established by the International Society for Cell & Gene Therapy (ISCT®) Mesenchymal Stromal Cell

committee [4] . MSCs used in this study showed typical spindle-shaped morphology and attachment to the plastic surface (Figure S3A). S-MSCs and BM-MSCs were both positive for classical MSC markers – CD90, CD73, CD44 (>96%), CD105 (>83%) and negative for hematopoietic stem cell markers – CD34 (<2.5%), CD45 (<0.1%), CD14 (<0.6%) (Figure S3B). S-MSCs and BM-MSCs have been shown to be able to differentiate into osteocytes, adipocytes and chondrocytes (Figure S3C).

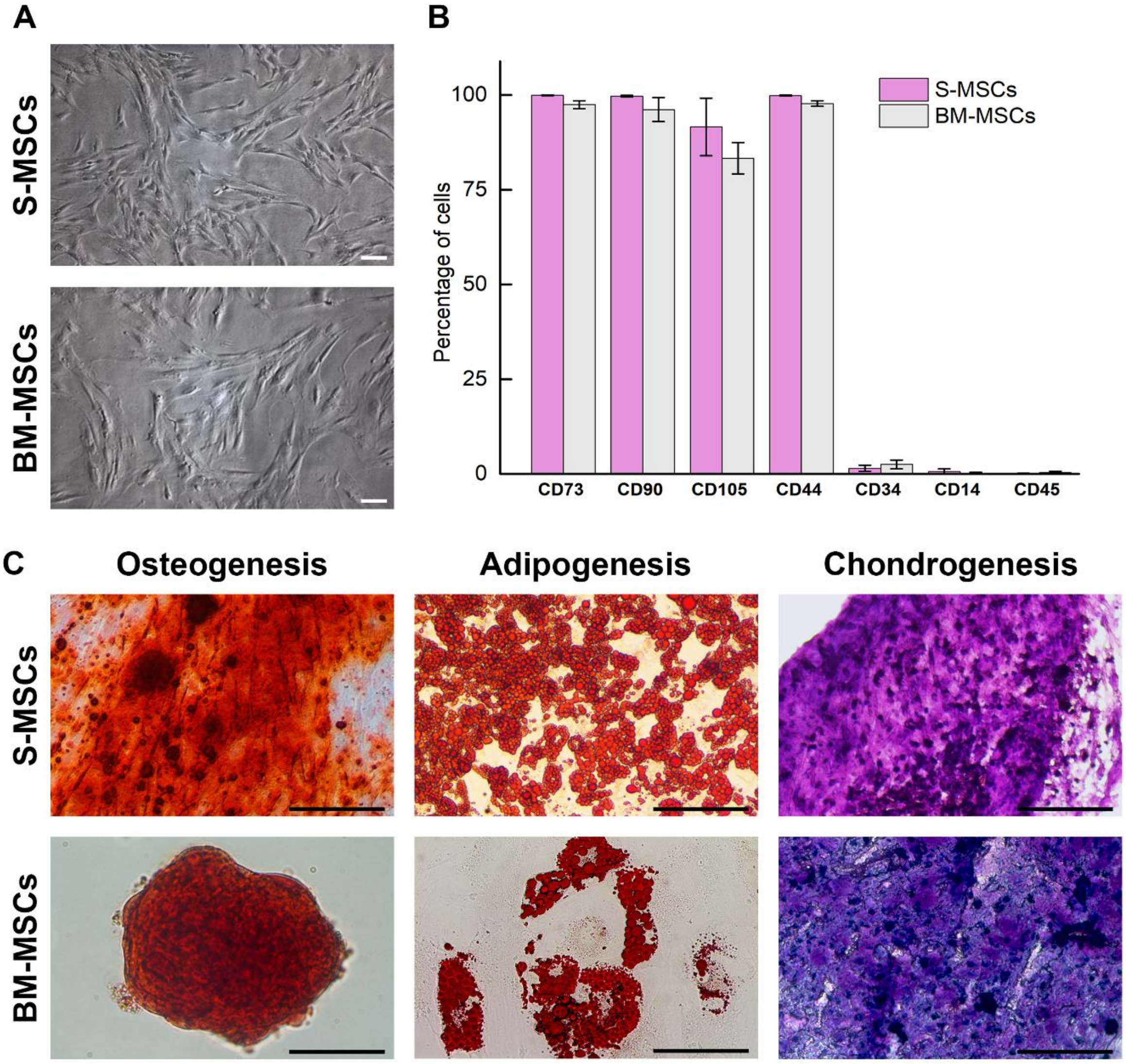

Figure S3. Characterization of S-MSCs and BM-MSCs. A – morphology of S-MSCs and BM-MSCs. B – expression of surface markers (CD73, CD90, CD105, CD44, CD34, CD14, CD45) in S-MSCs and BM-MSCs. Flow cytometry analysis. C – S-MSCs and BM-MSCs differentiation into osteogenic, adipogenic and chondrogenic lineage. Scale bar – 100 µm.

Migration study using Transwell migration assay

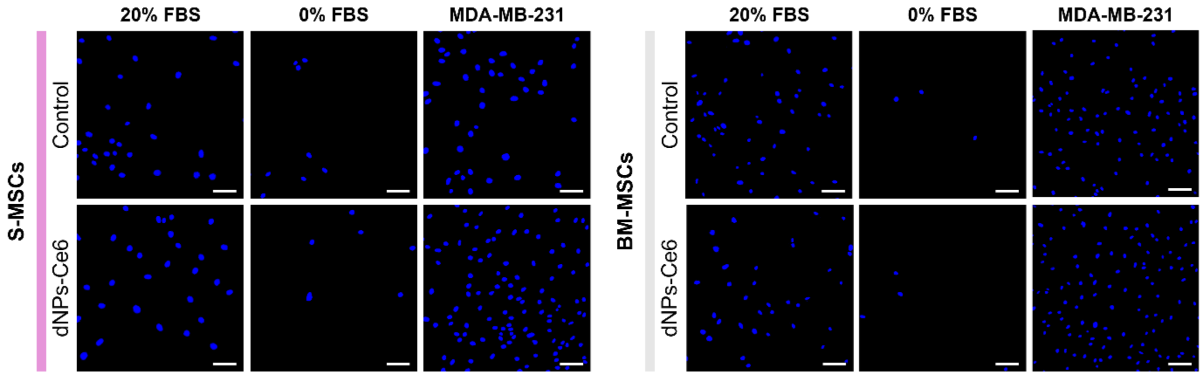

Figure S4. S-MSC and BM-MSCs migration towards 0% FBS, 20% FBS or MDA-MB-231 cells in the presence or absence of dNP-Ce6 complex. Blue – nuclei stained with Hoechst (λ_{ex} = 404 nm). Scale bar – 100 µm.

Photothermal effect

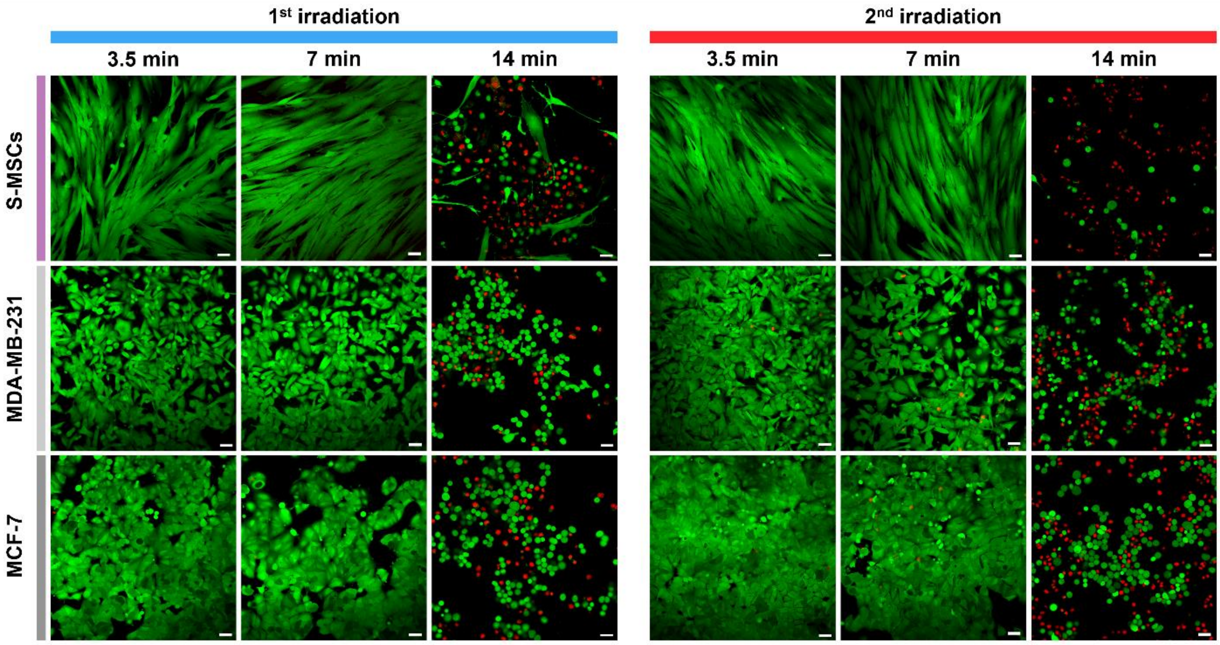

Figure S5. Photothermal effect on S-MSCs, MDA-MB-231 and MCF-7 cell lines. Cells were irradiated 1 or 2 times (24 h apart) with three different doses using a 980 nm laser (P_{980} =0.9 W). Green – live cells stained with calcein-AM (λ_{ex} = 488 nm), red – nuclei of dead cells stained with propidium iodide (λ_{ex} = 543 nm). Scale bar – 50 μm.

NIR imaging under 808 nm excitation

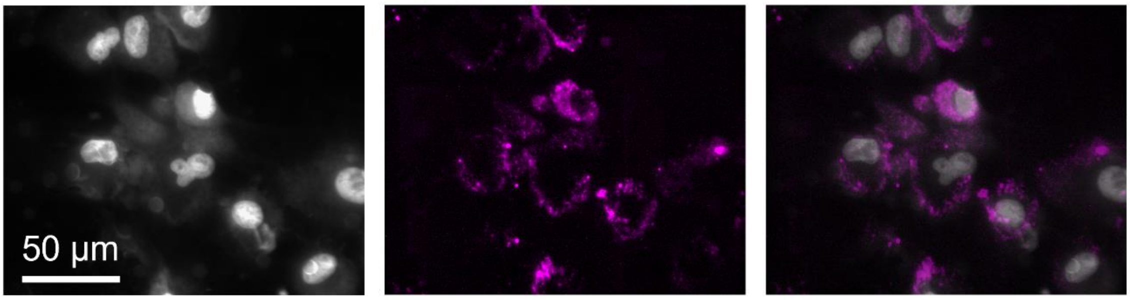

Figure S6. NIR imaging of dNP-Ce6 complex within MSCs after migration through 8 μm membrane. From left to right: visible fluorescence image (nuclei stained with Hoechst, λ_{ex} = 390 nm), NIR emission of dNP-Ce6 complex (λ_{ex} = 808 nm), and overlap of the two previous images. Scale bar – 50 μm.

References

(1) Jiang, G.; Pichaandi, J.; Johnson, N. J. J.; Burke, R. D.; van Veggel, F. C. J. M. An Effective Polymer Cross-Linking Strategy To Obtain Stable Dispersions of Upconverting NaYF4 Nanoparticles in Buffers and Biological Growth Media for Biolabeling Applications. *Langmuir* **2012**, *28* (6), 3239–3247. https://doi.org/10.1021/la204020m.
(2) Dapkute, D.; Pleckaitis, M.; Bulotiene, D.; Daunoravicius, D.; Rotomskis, R.; Karabanovas, V. Hitchhiking Nanoparticles: Mesenchymal Stem Cell-Mediated Delivery of Theranostic Nanoparticles. *ACS Appl Mater Interfaces* **2021**, *13* (37), 43937–43951. https://doi.org/10.1021/acsami.1c10445.
(3) Uzieliene, I.; Bironaite, D.; Bagdonas, E.; Pachaleva, J.; Sobolev, A.; Tsai, W.-B.; Kvederas, G.; Bernotiene, E. The Effects of Mechanical Load on Chondrogenic Responses of Bone Marrow Mesenchymal Stem Cells and Chondrocytes Encapsulated in Chondroitin Sulfate-Based Hydrogel. *International Journal of Molecular Sciences* **2023**, *24* (3), 2915. https://doi.org/10.3390/ijms24032915.
(4) Viswanathan, S.; Shi, Y.; Galipeau, J.; Krampera, M.; Leblanc, K.; Martin, I.; Nolta, J.; Phinney, D. G.; Sensebe, L. Mesenchymal Stem versus Stromal Cells: International Society for Cell & Gene Therapy (ISCT®) Mesenchymal Stromal Cell Committee Position Statement on Nomenclature. *Cytotherapy* **2019**, *21* (10), 1019–1024. https://doi.org/10.1016/j.jcyt.2019.08.002.
